# Genomics-driven drug repurposing and novel targets identification for sickle cell disease in Saudi patients

**DOI:** 10.3389/fbinf.2025.1671626

**Published:** 2025-12-02

**Authors:** Ali Alghubayshi, Mohammad A. Alshabeeb, Dayanjan Wijesinghe, Mohammed AlAwadh, Suad Alshammari, Khalifa Alrajeh, Mona A. Alkhairi, Imadul Islam, Ahmed Alaskar

**Affiliations:** 1 Department of Clinical Pharmacy, College of Pharmacy, University of Ha’il, Ha’il, Saudi Arabia; 2 Department of Pharmacotherapy and Outcomes Science, School of Pharmacy, Virginia Commonwealth University, Richmond, VA, United States; 3 Pharmaceutical Analysis Center, King Abdullah International Medical Research Center, Riyadh, Saudi Arabia; 4 King Saud bin Abdulaziz University for Health Sciences (KSAU-HS), Ministry of National Guard Health Affairs (MNGHA), Riyadh, Saudi Arabia; 5 Department of Pharmaceutical Chemistry, Faculty of Pharmacy, King Abdulaziz University, Jeddah, Saudi Arabia; 6 Department of Clinical Pharmacy, College of Pharmacy, Northern Border University, Rafah, Saudi Arabia; 7 Department of Pharmacy Practice, College of Clinical Pharmacy, King Faisal University, Hofuf, Saudi Arabia; 8 Department of Oncology, King Abdulaziz Medical City, Ministry of National Guard Health Affairs (MNGHA), Riyadh, Saudi Arabia

**Keywords:** sickle cell disease, drug repurposing, Saudi population, precision medicine, genome-wide association studies, pharmacogenomics

## Abstract

**Background:**

Sickle cell disease (SCD) is an inherited blood disorder characterized by chronic hemolysis, inflammation, and vaso-occlusive crises (VOC), leading to multiple complications and reduced life expectancy in affected individuals. Limited effective treatment options are currently available; however, recent genomic findings from underrepresented populations (Saudi Arabians) have offered new hope for predicting molecularly guided treatments. This study aimed to identify approved drugs suitable for repurposing based on their interactions with SCD-associated genetic variants and to discover novel druggable targets within genetic pathways linked to disease severity by utilizing genome-wide association study (GWAS) data from Saudi SCD patients.

**Methods:**

Bioinformatic pipelines were used to evaluate drug-gene interactions and identify potential therapeutic targets based on GWAS data derived from the Saudi population. Approved drugs were suggested for repurposing according to their interactions with genes known to impact SCD pathophysiology, using the Drug-Gene Interaction Database (DGIdb 5.0). New drug targets were also proposed by assessing the simulated binding pockets of gene products, using 3D protein structures from the Protein Data Bank (PDB) and the AlphaFold database. Molecules with higher druggability scores, as estimated by the DoGSiteScorer database, were predicted to have a higher success rate for new SCD treatment development.

**Results:**

Our analysis identified 78 approved medications with potential for repurposing in SCD; this list was narrowed to 21 candidates based on safety profiles and interactions with key genetic pathways. Among these, simvastatin, allopurinol, omalizumab, canakinumab, and etanercept were suggested as the most promising agents. Furthermore, novel drug targets encoded by olfactory receptor (OR) gene clusters (OR51V1, OR52A1, OR52A5, OR51B5, and OR51S1), TRIM genes, SIDT2, and CADM3 displayed high druggability scores.

**Conclusion:**

This study provides a robust framework for drug repurposing and novel drug discovery in SCD, particularly tailored to the Saudi population. The findings underscore the potential of leveraging genomic data to identify targeted therapies, offering a pathway to more personalized and effective treatments for SCD patients. Future clinical trials are essential to validate these findings and translate them into clinical practice.

## Graphical Abstract

**Figure:**
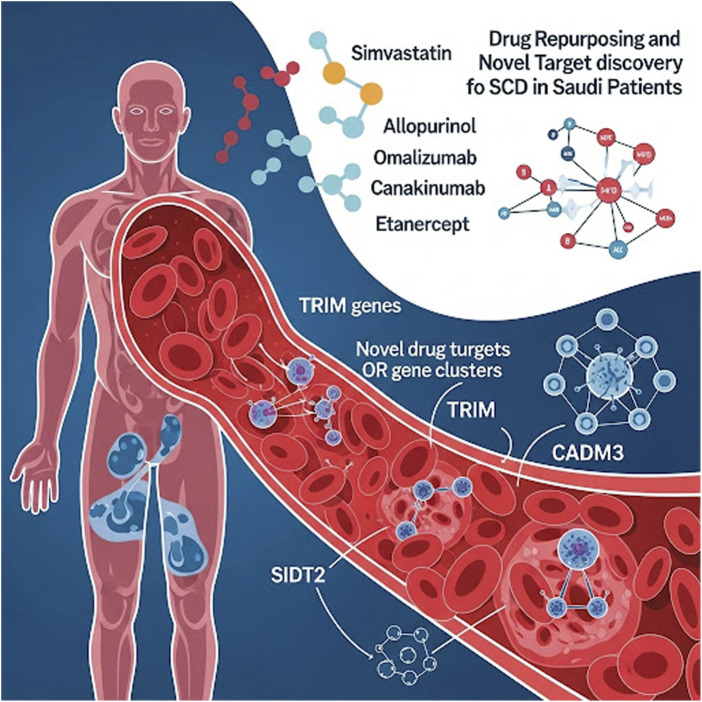

## 1 Introduction

Sickle cell disease (SCD) is a serious inherited hematological disorder caused by a pathological hemoglobin variant (*rs334* c.20 A>T) in the *HBB* gene, which induces rigid deformation or “sickling” of red blood cells (erythrocytes) (Inusa et al., 2019). This sickling leads to a range of downstream vascular complications, including vaso-occlusive episodes, thrombosis, and multiorgan infarctions, contributing to the clinical severity of SCD from early childhood onward (Inusa et al., 2019; Belisário et al., 2018). The severe nature of these complications is further highlighted by the reduced life expectancy among patients compared to unaffected individuals in the general population (Nnodu et al., 2021).

From a public health perspective, SCD imposes a substantial global burden. While some published estimates suggest there may be over 7 million individuals living with SCD worldwide (GBD, 2021 Sickle Cell Disease Collaborators et al., 2023), the World Health Organization (WHO) estimates that approximately 300,000 infants are born annually with the condition. Nigeria alone is estimated to have about 150,000 SCD births per year, making it the country with the highest incidence globally (Kumar and Bhattacharya, 2024). However, due to high childhood mortality rates in many endemic regions and underdiagnosis, particularly in sub-Saharan Africa and South Asia, the true number of affected individuals remains uncertain and is debated in the literature. Significant burdens also exist in India, the Americas, the Mediterranean, and the Middle East, though accurate epidemiological data is often limited by inadequate surveillance and historically high childhood mortality rates (Ware et al., 2017).

Reflecting this global burden, Saudi Arabia faces a notably high burden of SCD, with reported carrier frequencies that range up to 27%, while disease prevalence estimates of approximately 2.6% have been cited in the literature (Jastaniah, 2011; Bin Zuair et al., 2023). The indicated prevalence figures are not national averages and may vary substantially across different regions of the country, with higher rates observed in the Eastern and Southwestern provinces where SCD is most endemic. This substantial burden is likely exacerbated by high rates of consanguineous marriages (greater than 50%), which concentrate inherited disease variants (Jastaniah, 2011; el-Hazmi et al., 1995). Nearly 90% of high-risk couples (both carriers) proceeded with marriage, though the premarital screening program instructs the couples about these at-risk marriages (Jastaniah, 2011).

Despite the significant unmet medical need, the range of approved therapies for SCD remains remarkably limited. Originally, hydroxyurea was the only treatment approved by the US FDA for both adults and children with SCD. Since 2017, the therapeutic landscape has evolved, with the US FDA approving three new agents targeting distinct aspects of SCD pathophysiology, including: L-glutamine, crizanlizumab, and voxelotor (Kavanagh et al., 2022; Salinas et al., 2020). However, recent developments have highlighted the challenges in this field. Notably, as of September 2024, voxelotor has been voluntarily withdrawn globally due to safety concerns, and previously in May 2023, crizanlizumab was withdrawn from the European market due to lack of efficacy (pfizer, 2024; European Medicines Agency, 2023). While the European Medicines Agency (EMA) withdrew approval after reviewing the negative new STAND trial data showing no added benefit, the FDA continues to approve crizanlizumab usage, but is awaiting comprehensive review of the newer, conflicting data. These events underscore the complexities of SCD treatment and emphasize the critical importance of ongoing research, rigorous long-term safety monitoring, and continuous re-evaluation of approved therapies.

While bone marrow transplantation (BMT) offers a potential cure, its application is often hindered by significant challenges, including high costs, donor availability, and associated risks. The recent approval of the first cell and gene therapies, Casgevy and Lyfgenia, on December 8, 2023, marked a substantial advancement in SCD treatment (Leonard and Tisdale, 2024). However, these cutting-edge therapies remain inaccessible to many patients due to their extremely high costs. Casgevy is priced at $2.2 million USD per patient for a single treatment, while Lyfgenia costs $3.1 million USD per patient. These gene therapies are among the most expensive medicines ever approved and represent a significant economic challenge for both patients and health systems (Rueda et al., 2024).

This situation highlights the urgent need for further research into innovative therapies that offer safer, more effective, and affordable treatment options for SCD.

Furthermore, the complex genetic nature of SCD, coupled with recently identified disease-associated genetic variants from genome-wide association studies (GWAS), presents an ideal opportunity to guide both drug repurposing efforts and novel therapeutic development (Burt and Dhillon, 2013; Alghubayshi et al., 2025). Recent GWAS studies in Saudi populations—particularly by Alshabeeb et al. (2023) and Alghubayshi et al. (2025)—have begun to clarify the genetic landscape, showing that Saudi-specific variants are partly population-specific, with both unique and shared markers compared to African-origin populations. These findings indicate notable genomic differences due to the unique “Arab-Indian” haplotype prevalent in eastern Saudi Arabia, which differs in prevalence and clinical consequences from the African haplotypes (Benin, Senegal, Bantu, Cameroon). Drug repurposing represents a systematic methodology to propose new indications for currently marketed drugs based on their interactions with key genes that modulate cellular pathways and potentially ameliorate disease outcomes (Tragante et al., 2018). This computationally driven approach, which relies on *in silico* gene target analyses, has been used effectively to repurpose multiple drugs for various disease conditions (Pushpakom, 2024).

Our research focuses on repurposing existing drugs and discovering new molecules for SCD, which would then undergo clinical trials. Such advancements could revolutionize SCD management, enhancing scientific knowledge, technical capabilities, and clinical practice through the identification of novel genetic targets. These efforts have the potential to lead to groundbreaking treatments, setting new standards in the field and driving further innovation in personalized medicine.

In addition to the *HbS* variant, the severity spectrum of SCD manifests extensive clinical heterogeneity influenced by multiple genes (Kirkham et al., 2023). Previous genetic studies have uncovered polymorphisms that modulate different disease molecular pathways, including hemolysis, inflammation, oxidative stress, and vascular dysfunction (Kirkham et al., 2023; Pincez et al., 2022; Page et al., 2021). Examples include fetal hemoglobin (HbF)-regulating variants in *BCL11A* and *HBS1L-MYB*, as well as pro-inflammatory cytokine polymorphisms in *TNF-α* and *VCAM-1*, which are associated with an increased risk of stroke (Pincez et al., 2022). These genetic insights not only provide critical frameworks for repurposing existing drugs to target emergent SCD mechanisms but also pave the way for developing innovative, tailored therapies that could significantly improve patient outcomes (Pritchard et al., 2017).

The shortage of viable treatments for SCD has prompted the exploration of alternative strategies, such as pathway-based, phenotypic, or *in silico* drug repurposing, leveraging existing compound libraries or approved agents to target novel SCD disease mechanisms (Metaferia et al., 2022; Agu et al., 2023; Telen et al., 2019). Integrating genetic data from recent high-throughput genomic studies across diverse SCD patient populations enables the rational identification of putative druggable targets and prioritizes candidates for screening (Kirkham et al., 2023; Alshabeeb et al., 2023). However, most investigations have predominantly focused on African or African-American ancestries, with limited examination of other susceptible populations such as those in the Mediterranean and Middle Eastern regions (Driss et al., 2009).

Recently, our group uncovered Saudi-enriched genetic variants that drive thromboembolic complications in SCD patients, contrasting with markers identified in other cohorts (Alshabeeb et al., 2023). Additionally, findings from our current data have identified several loci within known genes linked to molecular pathophysiological pathways potentially influencing SCD severity, underscoring the need for tailored interventions (Alghubayshi et al., 2025). Expanding the characterization of population-specific molecular underpinnings through comprehensive genomics offers two key advantages: it uncovers novel therapeutic targets specific to Saudis and clarifies the heterogeneity in pathogenic mechanisms across the global SCD patient spectrum.

Therefore, in this study, we aimed to leverage findings from our GWAS analysis in conjunction with druggable target enrichment analysis, specifically focusing on Saudi SCD patients, to propose new SCD treatment options. Our overarching goal is to develop an informed, genetically guided roadmap that accelerates the development of innovative, population-tailored therapeutics for this historically overlooked SCD patient group. This holistic, molecular knowledge-based approach also seeks to evaluate and select the most promising druggable, non-targeted genes, thereby refining and expediting future drug discovery efforts.

## 2 Methods

### 2.1 The pipeline for drug repurposing and novel drug development

**FIGURE 1:**
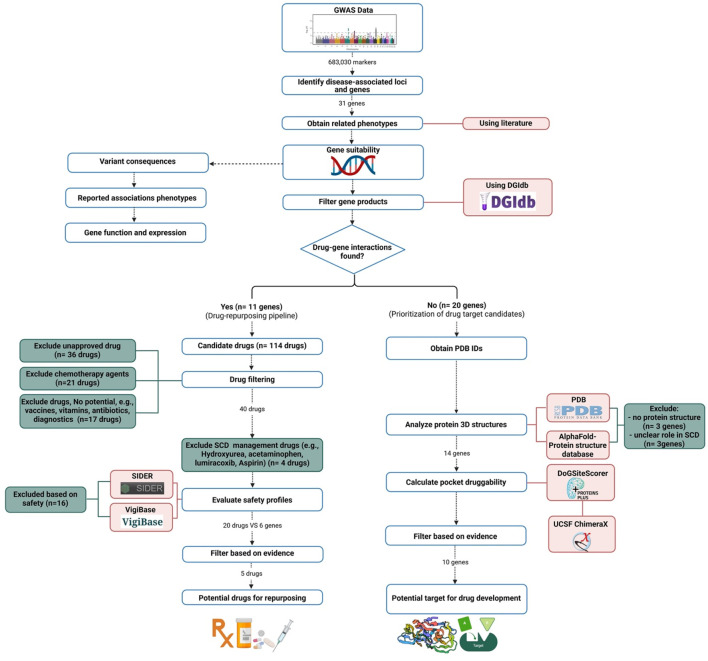
The pipeline for drug repurposing and novel drug target identification.

We utilized data derived from our previous Saudi SCD GWAS analysis (Alghubayshi et al., 2025) to assess the druggability of genes identified in Saudi SCD patients that may impact disease outcomes, aiming to establish their feasibility as therapeutic targets. Our analysis focused on identifying potential gene-drug interactions to pinpoint candidates for drug repurposing and to suggest new molecules to be developed as future treatment options. The analysis in this study followed a previously published pipeline (Figure 1) (Tragante et al., 2018).

### 2.2 Selection of SCD severity-associated genes

We focused on the reported 31 genes associated with SCD severity (Alghubayshi et al., 2025). These genes were selected based on their involvement in key SCD pathological pathways, including inflammation regulation, vascular function, endothelial modulation, and other disease-relevant biological processes. The selection criteria incorporated both pathway analysis of the identified genes and a comprehensive literature review, which demonstrated the functional relevance of these genes to SCD clinical phenotypes.

### 2.3 Drug-gene interaction analysis for drug repurposing

Gene products were filtered based on their potential to interact with medicinal drugs using the Drug-Gene Interaction Database (DGIdb) version 5.0, which consolidates data from multiple sources, including DrugBank and the Pharmacogenomics Knowledge Base (PharmGKB), to identify potential drug repurposing opportunities (Cannon et al., 2024). The database provides detailed annotation of drug-gene interactions and gene druggability, covering approved drugs and experimental compounds. Since gene products can interact with multiple drugs, we first excluded agents not clinically available (e.g., terminated or investigational drugs) due to their lack of immediate clinical applicability. Approved medications were then mapped to the genes of interest with known impact on disease pathways, excluding non-drug products.

During the initial filtration, we excluded terminated or withdrawn drugs. We prioritized medications targeting common SCD complications (e.g., inflammation, vascular dysfunction, immune dysregulation), as these pathways directly influence SCD severity. Diagnostic agents, vitamins, supplements, and superseded medications (replaced by more effective alternatives) were also excluded.

Candidate medications were further prioritized using predicted safety profiles. Using VigiBase (the largest global adverse event repository, https://www.vigiaccess.org/) (WHO, 2024), and the Side Effect Resource (SIDER 4.1) database (http://sideeffects.embl.de/) (Kuhn et al., 2016),we excluded drugs linked to severe adverse reactions. Chemotherapy agents and drugs with serious toxicities were excluded due to high-risk safety profiles. Agents posing a risk of hemolytic anemia were also removed to prevent exacerbating hemolysis in SCD patients. For comprehensive identification, we used the Search Tool for Interactions of Chemicals (STITCH) and the World Health Organization (WHO) databases to standardize drug nomenclature to match the International Nonproprietary Names (INN) and United States Adopted Names (USAN) and assign Anatomical Therapeutic Chemical (ATC) codes (Kuhn et al., 2016; Van Bever et al., 2014). We prioritized avoiding medications that could worsen SCD severity, especially those with risks of infection, cognitive impairment, or blood pressure instability, given SCD patients’ heightened vulnerability to these complications.

### 2.4 Novel drug development pipeline

Genes identified in our GWAS study (Alghubayshi et al., 2025), with no known interaction with approved drugs, were labeled as non-targeted genes. Selection criteria included: 1) variant consequences, 2) reported associations with SCD phenotypes, 3) gene function, and 4) expression in SCD-relevant tissues. Druggability scores were calculated to assess the likelihood of a compound interacting with a these gene products, based on the structural compatibility of their binding pockets with drug molecules.

Protein druggability was ranked using DoGSiteScorer (Volkamer et al., 2012), which analyzes Protein Data Bank (PDB) structures to evaluate physicochemical interactions and identify ligand-binding pockets (https://www.rcsb.org/) (Berman et al., 2007). DoGSiteScorer employs a difference Gaussian filter to detect potential pockets based solely on the 3D protein structures. Score ranges between 0 and 1, with values > 0.5 indicating potential druggability. This threshold was validated using 12 drug-targeted protein structures (scores: 0.68 to 0.88; Supplementary Table), enabling prioritization of candidate genes for drug development.

To address the absence of experimentally determined structures of some proteins in PDB, we used the AlphaFold database (Jumper et al., 2021), which provides highly accurate artificial intelligence (AI)-predicted structures. AlphaFold employs deep learning to predict protein structures with near-experimentally accuracy. Notably, its developers were awarded the 2024 Nobel Prize in chemistry for this breakthrough (Callaway, 2024). AlphaFold excels at predicting high-accuracy static structures of single proteins and, with newer versions (e.g., AlphaFold-Multimer, AlphaFold 3), can now model some protein complexes and small-molecule interactions, which is highly valuable for drug discovery and target prioritization. Its predictions have accelerated target identification and initial drug screening, especially for proteins lacking experimental structures (Desai et al., 2024). Combined with DoGSiteScorer, these tools enable identification of theoretical binding sites even without experimental structural data.

Predicted binding sites were visualized using UCSF ChimeraX V1.8 to generate 3D protein structure figures. Gene product expression was validated via the Human Protein Atlas (https://www.proteinatlas.org/) (proteinatlas.org, 2024), the Gene Expression Omnibus (GEO) of the National Center for Biotechnology Information (NCBI, https://www.ncbi.nlm.nih.gov/geo/) (Home - GEO - NCBI, 2024), or the Genotype-Tissue Expression (GTEX) portal (https://www.gtexportal.org/) (GTEx Portal, 2024). Genes with predominant expression in tissues unaffected by SCD were excluded (Supplementary Table).

## 3 Results

### 3.1 Promising candidates for drug repurposing

Out of the 31 genes suggested as modifiers for SCD phenotypes, 11 genes (*ACKR1, AGER, FCER1A, HLA-A, HLA-DQB1, HLA-DRB1, HLA-G, NOTCH4, RRM1*, *STIM1, and TRIM5*) showed the ability to interact with a total of 114 distinct drug molecules. Some of these drugs (n = 36) are not yet approved for clinical use and therefore excluded. The *HBG2* gene did not show any interaction with approved drugs. Among the remaining 78 drugs, chemotherapies (n = 21), vitamins and diagnostic agents (n = 2), vaccines (n = 3), and antimicrobials (n = 12) were excluded. The remaining medications (n = 40) were further filtered based on their safety profiles.

Consequently, we excluded anticonvulsants (n = 5), antidepressants (n = 5), antithyroid (n = 3), glucocorticoids, ticlopidine, and hydralazine due to their undesirable side effects. Hydroxyurea and analgesic agents (e.g., acetaminophen, aspirin, and lumiracoxib) were identified by our pipeline but were not included in the final suggested list, as they are routinely prescribed for managing SCD in clinical practice.

As a result, we narrowed down the list to the interactions between 20 medications and 6 genes, with 18 of these medications interacting with four *HLA* genes (*HLA-DQB1, DRB1*, *G*, and *A*). The remaining two medications (omalizumab and allopurinol) interact with two distinct genes (*FCER1A* and *NOTCH4*, respectively) [as shown in Table 1]. The most frequently detected therapeutic class was the immunosuppressants (n = 12), including adalimumab, canakinumab, infliximab, omalizumab, tocilizumab, etanercept, rilonacept, peginterferon alfa-2a and 2b, azathioprine, glatiramer, and anakinra. The second most common class was lipid-modifying agents (n = 7), which included fenofibrate and statins (pitavastatin, pravastatin, atorvastatin, simvastatin, rosuvastatin, and fluvastatin).

**TABLE 1:** Predicted drug-gene interactions identified in DGI database (V 5.0).

SNP^a^	Gene^a^ (n = 6)	Interacting drug (n = 20)	ATC code (s)	Interaction score
rs2494250	*FCER1A*	Omalizumab	R03DX05	0.94
rs2844806	*HLA-A*	Fenofibrate	C10AB05	0.15
		Peginterferon alfa-2a	L03AB11	0.13
		Peginterferon alfa-2b	L03AB10	0.12
rs3135006	*HLA-DQB1*	Infliximab	L04AB02	0.23
rs2395522	*HLA-DRB1*	Pitavastatin	C10AA08	0.48
		Azathioprine	L04AX01	0.06
		Pravastatin	C10AA03	0.09
		Atorvastatin	C10AA05	0.04
		Simvastatin	C10AA01	0.05
		Rosuvastatin	C10AA07	0.10
		Fluvastatin	C10AA04	0.20
		Tocilizumab	L04AC07	0.48
		Adalimumab	L04AB04	0.07
		Rilonacept	L04AC04	0.80
		Canakinumab	L04AC08	1.19
		Anakinra	L04AC03	0.27
		Etanercept	L04AB01	0.09
		Infliximab	L04AB02	0.23
		Glatiramer	L03AX13	0.51
rs2524035	*HLA-G*	Simvastatin	C10AA01	1.05
rs3132946 rs3132940 rs9267898 rs3096702	*NOTCH4*	Allopurinol	M04AA01	0.58

^a^ Genetic variants and mapped genes associated with sickle cell disease severity, as identified in our genome wide association study of Saudi patients (Alghubayshi et al., 2025).

Ultimately, immunomodulators—including monoclonal antibodies—and statins were proposed as the most suitable candidates for repurposing to manage SCD severity, based on their interactions with key genes involved in critical SCD pathological pathways and supported by published evidence (Alghubayshi et al., 2025; Kirkham et al., 2023). Specifically, three immunomodulators, each acting on different gene targets (omalizumab targeting *FCER1A,* and canakinumab as well as etanercept targeting *HLA-DRB1*), along with a statin (simvastatin, which interacts with both *HLA-G* and *HLA-DRB1*), demonstrated high drug-gene interaction score. Additionally, the antihyperuricemic agent allopurinol, which targets *NOTCH4*, was identified as a promising repurposing candidate.

### 3.2 Druggability analysis of non-targeted loci

Among the 31 genes suggested by our GWAS study (Alghubayshi et al., 2025), 11 were shortlisted as drug repurposing candidates, while the remaining 20 genes were evaluated for chemical structures and druggability. Three genes (*OR10J8P, OR10J9P, and HBBP1*) were excluded due to a lack of available protein structures. Additionally, *POC5* was excluded because of its unclear role in SCD pathology, and *SCAND3* and *MMP26* were excluded due to predominant expression in non-relevant tissues (e.g., testis and endometrium).

**FIGURE 2:**
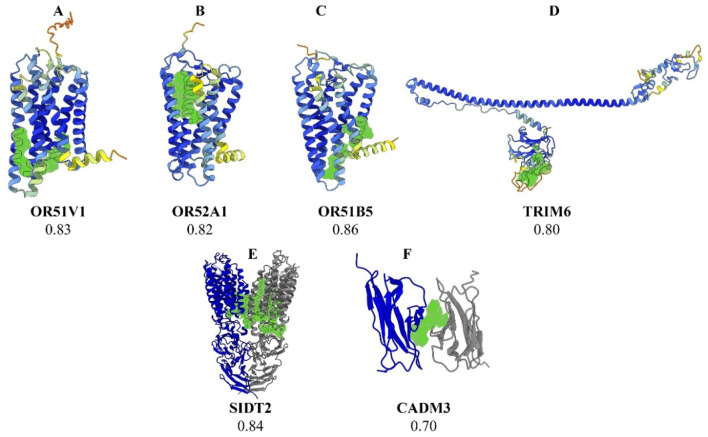
Three-dimensional structural representations of selected protein targets. Legend: **(A)** *OR51V1*, **(B)** *OR52A1*, **(C)** *OR51B5*, **(D)** *TRIM6*, **(E)** *SIDT2*, and **(F)** *CADM3*. The predicted optimal druggable binding site is shown in green for each protein. The protein backbone is colored by AlphaFold pLDDT confidence: dark blue (90–100, very high), light blue (70–90, confident), yellow (50–70, low), and orange (<50, very low). For SIDT2 **(E)** and CADM3 **(F)**, gray ribbons denote additional chains/subunits shown for context and are not colored by pLDDT.

Fourteen prioritized gene targets (*CADM3, HBD, HBG2, HBE1, MPTX1, OR51B5, OR51S1, OR51V1, OR52A1, OR52A5, SIDT2, TRIM22, TRIM34,* and *TRIM6*) demonstrated druggability scores ranging from 0.7 to 0.87, [See Table 2 for detailed descriptions of these molecules]. The olfactory receptor (*OR*) gene cluster (*OR51B5, OR51S1, OR51V1, OR52A1,* and *OR52A5*), and the Tripartite Motif gene family (*TRIM6, TRIM22*, and *TRIM34*), emerged as notable candidates. Further details for all screened molecules are provided in the Supplementary Table. Notably, the *OR* gene cluster—especially *OR51V1, OR51A1*, and *OR51B5—* has been strongly associated with SCD phenotypes in the literature. *TRIM6*, *SIDT2*, and *CADM3* were particularly recommended based on their high expression in SCD-relevant tissues and their potential roles in related complications. Structural visualizations and druggability scores for these leading protein candidates are presented in Figure 2.

**TABLE 2:** Most suitable drug targets based on predicted pocket interactions utilizing DogSiteScorer database.

SNP	Gene (n = 14)	Pocket score	PDB code	Alphafold code
rs147062602 rs10838058 rs10837853 rs78253695 rs180750244	*OR51B5*	0.86	----	AF-Q9H339-F1
rs12361955	*OR51S1*	0.84	----	AF-Q8NGJ8-F1
rs7933549	*OR51V1*	0.83	----	AF-Q9H2C8-F1
rs112098990	*OR52A1*	0.82	----	AF-Q9UKL2-F1
rs2472530	*OR52A5*	0.82	----	AF-Q9H2C5-F1
rs3740999 rs11038294 rs12272467	*TRIM6*	0.8	----	AF-Q9C030-F1
rs67573252	*TRIM22*	0.87	----	AF-Q8IYM9-F1
rs2342380	*TRIM34*	0.82	2EGP	----
rs10535646	*SIDT2*	0.84	7Y68	----
rs3845624	*CADM3*	0.7	1Z9M	----
	*MPTX1*	0.81	----	AF-A8MV57-F1
rs2071348	*HBD*	0.84	1SHR	----
rs2213170 rs7130110 rs2213169	Near *HBE1*	0.85	1A9W	----
rs2236794	*HBG2*	0.88	7QU4	---

^a^ Genetic variants and mapped genes associated with sickle cell disease severity, as identified in our genome-wide association study of Saudi patients (Alghubayshi et al., 2025).

## 4 Discussion

Applying genetic knowledge to enable the repurposing of drugs has emerged as a promising and innovative strategy in the field of pharmacology. In 2021, the U.S. Food and Drug Administration (FDA) approved approximately two-thirds of new medications based on genetic insights (Kang et al., 2024; Ochoa et al., 2022). Previous analyses suggest that drugs targeting genes supported by robust human genetic evidence have a higher probability of progressing successfully through clinical development and obtaining regulatory approval compared to drugs without such genetic support (Kirkham et al., 2023). However, approval is determined by a complex interplay of factors, including clinical trial design, population diversity, rigorous endpoint selection, safety and efficacy data, as well as post-marketing experience. While genomic support can strengthen the biological rationale and target validation underlying drug development, it does not alone guarantee success. The overall likelihood of approval is ultimately dependent upon integrated consideration of these multifaceted regulatory, scientific, and clinical parameters (Nelson et al., 2015).

The development and application of advanced genetic analysis approaches such as GWAS and next-generation sequencing (NGS) [e.g., whole-genome sequencing (WGS) and whole-exome sequencing (WES)] have significantly deepened our understanding of the genetic underpinnings of complex traits (Ochoa et al., 2022; Ullah, 2025; Ku et al., 2010).This progress has facilitated the identification of promising drug targets through the discovery of causative genetic variants (Reay and Cairns, 2021). However, effectively translating these genetic discoveries into clinical practice remains challenging, particularly for conditions like SCD with diverse phenotypes influenced by multifactorial genetic associations. The use of already authorized medications for new applications at more affordable costs is increasingly recognized as a valuable and expanding approach (Tragante et al., 2018; Kang et al., 2024; Drug Repurposing DR, 2024). Several previous studies have utilized an *in silico* pipeline to suggest potential drug candidates and identify target molecules for treating various medical conditions by leveraging findings from publicly available GWAS analyses (Tragante et al., 2018; Nanda et al., 2020; Xu et al., 2022). Different GWAS databases are available and can be used to understand the genetic basis of selected diseases. Therefore, it is possible to translate identified signals into therapeutic targets using genomic-based approaches and a well-established pipeline (Tragante et al., 2018). In our project, we adopted a similar methodology to propose novel drug treatment options that could potentially target specific genes known to impact outcomes in SCD patients.

In a previous GWAS study, we identified multiple genes that play a significant role in SCD and its complications (Alghubayshi et al., 2025). Furthermore, the current study aimed to maximize the utility of existing data to translate genetic knowledge into clinical care for SCD. Our pipeline detected the genes targeted by hydroxyurea (RRM1) and the analgesics (HLA-DQB1 and HLA-DRB1) which served as benchmarks to validate the pipeline’s performance (more details are shown in the Supplementary Material). The successful identification of these medications attests to the robustness of the pipeline. These therapeutic drugs were excluded from further evaluation because they are already indicated for SCD, rather than toxicity concerns. A key consideration in our repurposing pipeline was the systematic evaluation of both therapeutic potential and safety profiles across the 78 candidate drugs for SCD management. Safety considerations were rigorously applied at every filtration and drug prioritization step. In addition to hydroxyurea, other chemotherapy agents were excluded too based on their documented association with serious adverse reactions, including myelotoxicity, secondary malignancy, hepatotoxicity, cardiotoxicity, and profound immunosuppression (WHO, 2024; Kuhn et al., 2016). These risks are particularly critical in vulnerable populations such as patients with SCD. All candidate drugs identified by the pipeline, including those not advanced for primary analysis (e.g., chemotherapy agents), are presented in the Supplementary Material. This enables clinicians and researchers to transparently evaluate all mechanistically plausible agents and to design tailored clinical trials that weigh both benefit and risk according to patient-specific needs, emerging evidence, and evolving standards of care. Exclusion of some agents does not preclude their future consideration. Notably, recent studies of non-cytotoxic, low-dose or oral epigenetic modifiers such as decitabine (plus tetrahydrouridine) have shown promise, safely inducing fetal hemoglobin (HbF) and improving SCD metrics without significant marrow suppression or classic chemotherapeutic risks (Blood, 2024). These developments suggest that specific chemotherapeutic agents, when optimized for safety, should remain under consideration as part of a broader SCD therapeutic landscape—particularly for refractory or severe cases.

Twenty approved agents were identified as having strong affinity for interact with six genes modulating various SCD phenotypes. The chosen drug candidates are classified into lipid-modifying and immunomodulatory agents, with the potential to modulate multiple pathways in SCD, including endothelial function, sickling, hemolysis, and inflammation (Chowdhury et al., 2023). Based on unique criteria, the filtering process identified five highly promising candidates for repurposing: simvastatin, allopurinol, canakinumab, and etanercept—all of which had previously been involved in small experiments and clinical trials on SCD—as well as omalizumab. In addition to simvastatin, other statins were also identified, such as atorvastatin, pravastatin, fluvastatin, rosuvastatin, and pitavastatin, which have also shown promise for SCD management due to their anti-inflammatory properties. These agents downregulate the transcription factor NF-_K_B, inhibit the expression of key pro-inflammatory cytokines such as TNF and IL1B, and enhance endothelial function by restoring nitric oxide (NO) bioavailability and reducing the expression of adhesion molecules, collectively contributing to vascular protective effects (Adam and Hoppe, 2013; Bereal-Williams et al., 2012). Notably, the JUPITER trial, a key intervention study evaluating rosuvastatin, demonstrated the immunomodulatory potential of statins, with rosuvastatin significantly reducing C-reactive protein (CRP) levels by approximately 37%, further supporting the role of statins in mitigating inflammation-related complications (Ridker et al., 2008).

Simvastatin was suggested as the optimal candidate among all statins due to its superior interaction score and clinical relevance. While other statins interact with *HLA-DRB1* only, which encodes a major histocompatibility complex protein, simvastatin uniquely interacts with both *HLA-DRB1* and *HLA-G*. These proteins are integral to immune responses that exacerbate SCD complications, such as vaso-occlusive crises and organ damage (Wong et al., 2022; Tamouza et al., 2002; Ma et al., 2008; Martins et al., 2022). Furthermore, simvastatin has shown a greater capacity to reduce von Willebrand factor (vWF) levels, underscoring its crucial role in managing SCD-associated hypercoagulability (Akaba et al., 2020; Sahebkar et al., 2016).

A clinical study in a small SCD cohort (n = 26) demonstrated that short-term simvastatin administration significantly improved nitric oxide bioavailability by approximately 52% (p = 0.01) and suppressed systemic inflammatory biomarkers such as CRP and IL-6, both of which are associated with increased vascular dysfunction risks in SCD (Hoppe et al., 2011). Moreover, a pilot study on 19 SCD patients reported that simvastatin reduced the occurrence of vaso-occlusive pain by 85% (p = 0.0003) and decreased the use of analgesics by 67% (p = 0.003). It also showed a significant reduction in circulating CRP, soluble (s) E-selectin, intercellular adhesion molecule 1 (sICAM-1), sICAM-3, and vascular endothelial growth factor, with the most pronounced effects observed in patients concurrently receiving hydroxyurea (Hopp et al., 2017). While the cited studies of statins in SCD report promising reductions in inflammatory biomarkers and clinical pain episodes, the trial sizes were modest (n = 19–26), which impact robustness and generalizability. Thus, current evidence should be viewed as preliminary and highlights the need for well-powered randomized controlled trials to more clearly define the independent effects of statin therapy in SCD.

*In vitro* studies further underscore simvastatin’s potential, showing a 1.9-fold increase in fetal hemoglobin expression and a 30%–35% reduction in irreversibly sickled cells under hypoxic conditions (Xi et al., 2024). Based on this robust body of evidence and specific criteria, we recommend simvastatin as the ideal drug for repurposing in SCD, although other statins may also offer benefits depending on individual patient factors. Further research, including large randomized controlled trials, is warranted to fully elucidate simvastatin’s therapeutic potential in this context.

Accumulating clinical evidence indicates that statins (e.g., simvastatin, atorvastatin) are generally well tolerated in SCD, with minimal risk. Moreover, statins are widely available, affordable, and suitable for chronic, home-based administration, which supports their risk–benefit profile for long-term SCD management (Hopp et al., 2017). It is important to note that statin-induced myopathy, while generally rare, may have increased relevance in SCD due to the higher prevalence of renal or hepatic comorbidities (Ademi et al., 2025). Overlap with SCD musculoskeletal symptoms can also hinder timely detection of toxicity. Although current pilot studies did not observe excess myopathy, sample sizes and follow-up duration were limited (Hoppe et al., 2011). We therefore recommend routine monitoring of creatine kinase levels and consideration of pharmacogenetic testing (e.g., SLCO1B1 genotypes) to mitigate risk and individualize therapy (Choi et al., 2025).

Another suggested repurposing candidate is allopurinol, which targets products of the *NOTCH4* gene. This gene is a member of the *NOTCH* family, well-known for its role in regulating endothelial function and exerting anti-inflammatory effects, as well as influencing hematopoiesis, all of which may contribute to alleviating disease severity (López-López et al., 2021; Zhou et al., 2022). In the Saudi SCD dataset, we identified the marker rs3132946 in *NOTCH4*, which has been previously reported as a marker linked to interstitial lung diseases (ILD) (Fingerlin et al., 2013). This condition is notably common in SCD patients, with 74% of adults in a large prospective cohort study exhibiting a restrictive pattern on pulmonary function tests (Field et al., 2012). While not all adults with SCD show the common features of sickle cell lung disease (SCLD), this restrictive pattern remains the most consistent clinical manifestation, underscoring its significant impact on this population.

Allopurinol, a well-established xanthine oxidase inhibitor long-used to treat hyperuricemia in patients with gout or tumor lysis syndrome (Adeyinka and Bashir, 2024), has demonstrated broader vascular benefits. It can protect vascular tissue from oxidative stress and repetitive reperfusion injury, both of which are critical components of SCD pathology (Pritchard et al., 2005). By inhibiting the production of reactive oxygen species (ROS), allopurinol preserves nitric oxide availability, thereby supporting vascular relaxation and function (Kelkar et al., 2011). Preclinical studies in sickle cell models show mixed results; while one study reports no significant effect on cell adhesion, another showed improved blood flow and reduced leukocyte recruitment upon exposure to allopurinol (Wood et al., 2005; Kaul et al., 2004). Given the oxidative stress and endothelial dysfunction observed in SCD, further exploration of allopurinol’s therapeutic potential, particularly in combination with other agents, is warranted. However, careful consideration of its side effects, including hypersensitivity and rare severe reactions, is crucial. Even in populations where the HLA-B*5801 allele is not highly prevalent, such as the Saudi population (Dashti et al., 2022), it is still important to genotype for HLA-B*5801 to mitigate the risk of hypersensitivity reactions when prescribing allopurinol (Dean et al., 2012).

Monoclonal antibodies and related agents (e.g., omalizumab, canakinumab, etanercept), although requiring regular injections and incurring higher costs, have demonstrated safety and efficacy in reducing inflammation and acute complications in SCD. Reported adverse outcomes, such as increased infection risk, are generally manageable and are outweighed by their capacity to target severe SCD manifestations in select cases (Yessayan et al., 2022). Their use has shown acceptable safety in recent clinical settings for SCD and other hyperinflammatory states, with no increase in serious adverse outcomes (Rees et al., 2022). Omalizumab was initially approved in 2003 by the US FDA for the treatment of moderate to severe persistent asthma, with expanded indications in subsequent years (Drugs.com, 2024). This medication, which targets the FCER1A gene product, has emerged as one of the top candidate repurposing drugs. This recombinant immunoglobulin G (IgG) is a monoclonal antibody that selectively binds to free IgE, thereby attenuating allergic asthma. By preventing IgE from interacting with high-affinity Fcε receptors on effector cells such as mast cells, omalizumab modulates downstream pro-inflammatory signaling that leads to airway inflammation and may also potentially reduce coagulation abnormalities associated with disease activity (Kaplan et al., 2017).

In the Saudi SCD cohort, the marker rs2494250 in *FCER1A* was detected. This variant was previously reported as significantly associated with elevated levels of inflammatory biomarkers, including CCL2/MCP-1 (Benjamin et al., 2007). Furthermore, studies have reported a higher prevalence of IgE among Saudis with SCD, potentially increasing the risk of acute chest syndrome (ACS). Notably, ACS accounts for 25% of ICU admissions and 28.5% of deaths among SCD patients, with particularly high rates observed in individuals from Al-Hasa region (Al-Suliman et al., 2006; Alhaj et al., 2023; An et al., 2011). Thus, further clinical studies in SCD patients are warranted to confirm omalizumab benefits in mitigating this disease complication.

Our pipeline has also identified other biological disease-modifying drugs targeting the *HLA-DRB1* gene. Canakinumab and etanercept were suggested as favorable candidates for repurposing in SCD management. Canakinumab can selectively target IL-1β, a cytokine with a central role in the inflammatory process, and may contribute to modulating disease pathways in SCD (Salinas et al., 2020; Awojoodu et al., 2014).

Moreover, canakinumab has been studied on 49 children and young adults with SCD in a recent double-blind, randomized study (Salinas et al., 2020; Rees et al., 2022). It demonstrated a tolerable safety profile, in contrast to other monoclonal antibodies that are often associated with a high risk of infection (Fernández-Ruiz and Aguado, 2018; Contejean et al., 2023). Although the randomized, placebo-controlled trial of canakinumab in children and young adults with SCD failed to meet its primary endpoint of reducing daily pain compared to baseline, several secondary outcomes showed potential benefits. Specifically, lower levels of inflammatory biomarkers, reduced hospitalization rates, and improvements in patient-reported outcomes, such as fatigue and school or work absenteeism, were observed. These findings suggest that while pain reduction was not achieved, canakinumab may still offer ancillary advantages in managing other aspects of SCD, but further research is needed to confirm its clinical utility. On the other hand, etanercept, a TNF-α receptor blocking agent, may halt inflammatory signaling initiated by TNF-α. Episodes of ischemia-reperfusion injury that commonly lead to vascular dysfunction and subsequent vaso-occlusion in SCD, may be minimized with etanercept therapy (Telen et al., 2019). Long-term exposure of mice with SCD to etanercept resulted in a reduction of acute disease manifestations, including vaso-occlusion, responses to pain stimuli, leukocytes, and inflammatory biomarkers (Solovey et al., 2017). In addition, two case reports of patients with rheumatoid arthritis and SCD were treated successfully and safely with etanercept (Adelowo and Edunjobi, 2011). This evidence supports the need for conducting clinical trials to assess etanercept’s outcomes in SCD patients.

These suggested agents show promising potential in managing SCD severity and offer diverse features, including different routes of administration and cost advantages, which could facilitate their clinical adoption. For instance, simvastatin and allopurinol are widely used, available as oral formulations, and offered as affordable generics, which enhances their accessibility for long-term use (Al-Suliman et al., 2006; Drugs.com, 2024a; Drugs.com, 2024b). In contrast, monoclonal antibodies also impact disease severity pathways; however, they require subcutaneous injections, often administered during hospital visits every 4–8 weeks, making them less convenient. Additionally, their higher costs compared to oral medications may limit accessibility (An et al., 2011; Drugs.com, 2024c; Drugs.com, 2024d). Thus, oral agents offer greater convenience as they can be taken at home daily, potentially improving adherence, especially in chronic conditions like SCD. While injectable biologics may be more potent, they present challenges related to frequent healthcare interactions, administration, and higher costs. Strategically selecting these medications based on patient compliance and healthcare setting considerations may provide valuable therapeutic options for managing SCD complications.

The complex pathophysiology of SCD necessitates targeting multiple possible disease mechanisms. Thus, a multi-drug approach is suggested to address factors such as fetal hemoglobin modulation, cell adhesions and sickling, inflammation, ischemia/reperfusion, oxidative stress, coagulation, and free heme toxicity. To achieve this, a combination of drug therapies with different mechanisms is warranted (Telen et al., 2019).

In exploring new potential drug targets, we successfully identified the druggability pocket in several previously non-targeted gene products within this pipeline. We strongly recommend *OR* gene clusters as novel targets, particularly *OR51V1*, *OR52A1*, and *OR51B5*. Important mutations in these genes—such as rs7933549 (missense), rs112098990 (frameshift), and rs147062602 (frameshift), respectively—are commonly carried by SCD individuals and are associated with defects in protein function and subsequent degradation. As shown in the GTEx portal, this gene cluster is notably expressed in whole blood and erythroid cells near the β-globin cluster, which plays a regulatory role in hemoglobinopathies (Bulger et al., 2000; Yang et al., 2007; Feingold et al., 1999). Although these are pseudogenes—non-functional DNA segments that do not directly contribute to phenotypic traits—they may play roles in regulating gene expression and function. Numerous genetic variants mapped to these gene products have been reported in the literature with multiple SCD phenotypes from previous GWAS and large meta-analyses, providing strong evidence of *OR* genes involvement in hematological parameters and SCD complications (Kirkham et al., 2023; Alshabeeb et al., 2023). The reported traits associated with various mutations in the *OR* genes include level measurements of hemoglobin, hematocrit, erythrocyte count, mean corpuscular volume, red blood cell distribution width, mean corpuscular hemoglobin concentration, mean reticulocyte volume, fetal hemoglobin level, leukocyte count, monocytes, neutrophils, CRP, and platelet levels. Clinical complications such as hemolysis, thromboembolism, ischemia, and cardiomyopathy in SCD have also been previously linked to these genes (Yang et al., 2007; Feingold et al., 1999; Chen et al., 2018).

The pipeline also suggests *TRIM 6*, *SIDT2*, and *CADM3* as novel, promising therapeutic targets. TRIM family proteins, including TRIM6, TRIM22, and TRIM34, are involved in innate immune modulation, cell cycle progression, and transcriptional regulation, suggesting they may influence inflammation, oxidative stress, hematopoietic stem cell differentiation, and immune responses (Uchil et al., 2013). These processes are critical to SCD pathophysiology and its complications. Furthermore, the *SIDT2* gene product may contribute to SCD-related outcomes, as its dysregulation is linked to several metabolic traits associated with hepatic disorders, cardiovascular dysfunctions, and SCD-nephropathy (Qian et al., 2023; Geng et al., 2021). SIDT2 is implicated in lysosomal function; its disruption may lead to defective cellular waste processing and metabolic imbalances, exacerbating organ dysfunction and complications often seen in SCD patients. Meanwhile, dysregulation of *CADM3*, which is involved in epithelial proliferation and cell cycle adhesion (Estelius et al., 2019), could impair bone marrow niche integrity and disrupt normal hematopoiesis, potentially influencing the production and maturation of erythroid and other blood cells. Such alterations in hematopoietic processes may exacerbate anemia, increase circulating immature cells, or modify immune responses, all of which are critical contributors to the heterogeneity and severity of SCD clinical manifestations (Jarczak et al., 2025). Each of these targets has a high druggable score, ranging from 0.7 to 0.86, highlighting them as strong candidates for future drug development.

### 4.1 Limitation

While the current study presents a promising framework to repurpose approved drugs as well as identify new drug targets for managing SCD severity, several limitations should be considered. First, our approach relies in part on *in silico* predictions and bioinformatic analyses, which, while powerful, cannot fully capture the complexities of drug-target interactions *in vivo*. This was partially addressed by the availability of various animal and small clinical trials that support our findings, yet larger, prospective clinical trials remain necessary. Additionally, the druggability scores that were used to prioritize the candidate targets for discovering new drug molecules were derived from computational models, which may not accurately reflect true biological conditions.

While AlphaFold represents a transformative advance in protein structure prediction, important limitations exist when using its models for druggability analyses such as with DoGSiteScorer. AlphaFold is trained primarily on apo-protein structures and does not account for ligand, cofactor, or complex-induced conformational changes, its predictions may not always reflect the true geometry of ligand-binding pockets or active sites. In particular, flexible regions, loops, and inducible binding sites—crucial for drug discovery—may be less accurately modeled than in experimentally resolved structures. Therefore, all computational predictions of pocket druggability from AlphaFold models should be interpreted as hypotheses, and high-confidence targets should be prioritized for experimental validation by X-ray crystallography or related structural biology methods before advancing toward drug development (Karelina et al., 2023). Furthermore, the absence of direct clinical evidence concerning some of the proposed targets, such as *SIDT2* and *CADM3*, in relation to SCD outcomes underscores the need for further experimental validation.

### 4.2 Conclusions

Previous analyses indicate that drugs targeting genes with robust human genetic evidence, such as findings from GWAS, have an enhanced probability of gaining clinical approval compared to those lacking such support. However, the overall likelihood of approval depends on a multitude of additional factors, rather than genetic evidence alone. Utilizing an established pipeline enabled us to identify several promising candidates for drug repurposing, notably statins, immunomodulatory agents, and allopurinol. Additionally, novel targets—specifically *OR* and *TRIM* gene families—were identified for potential drug development to mitigate SCD severity and complications, with a focus on the Saudi population. The selection of these gene targets was supported by high pocket druggability scores and their involvement in key molecular pathways underlying the disease. These findings not only enhance our understanding of the genetic basis of SCD but also pave the way for more targeted and effective therapeutic approaches. A logical roadmap for translating these findings would begin with targeted *in vitro* studies to confirm the functional impact of identified drug-gene interactions and novel targets, followed by *in vivo* validation to assess therapeutic efficacy and safety. Promising candidates can then advance to early-phase clinical trials to evaluate clinical feasibility, optimal dosing, and patient response, paving the way for population-specific, genetically guided therapies for SCD. Future clinical trials are essential to validate these computational predictions and translate them into actionable therapies that can improve outcomes for SCD patients.

## Data Availability

The datasets presented in this study can be found in online repositories. The names of the repository/repositories and accession number(s) can be found in the article/Supplementary Material.
