# Supplementary material for: Genomics-driven drug repurposing and novel targets identification for sickle cell disease in Saudi patients
Source: Front Bioinform. 2025 Dec 2;5:1671626. doi: 10.3389/fbinf.2025.1671626 (PMC12705626; doi:10.3389/fbinf.2025.1671626)
Supplement: Supplementary file 1 [file Table2.docx]

| Supplementary Table: Gene Product 3D Structure codes Predicted by Experimental and Artificial Intelligence (AI)-Based Methods with Corresponding Drug Scores and Highest Expression Tissue Sites | | | | |
| --- | --- | --- | --- | --- |
| **SNP (n=27)** | **Genes (n=20),**  **with no drug interaction** | **PDB Code**  (**druggability score)** | **Alpha fold code**  **(Druggability score)** | **Top Tissue expression Sites** |
| rs7933549 | *OR51V1* | x | AF-Q9H2C8-F1  (0.83) | Whole blood |
| rs112098990 | *OR52A1* | x | AF-Q9UKL2-F1  (0.82) | Whole blood |
| rs2472530 | *OR52A5* | x | AF-Q9H2C5-F1  (0.82) | Whole blood |
| rs147062602  rs10838058  rs10837853  rs78253695  rs180750244 | *OR51B5* | x | AF-Q9H339-F1  (0.86) | Adipose tissues and whole blood |
| rs12361955 | *OR51S1* | x | AF-Q8NGJ8-F1  (0.84) | Whole blood |
| rs6687840  rs4446959 | *OR10J8P, OR10J9P* | — | — | *OR10J8P*: Brain and whole blood  *OR10J9P*: Testis |
| rs3845624 | *CADM3,*  *MPTX1* | 1Z9M  (0.7) | --- | *CADM3*: Brain  *MPTX1*: Adipose tissues |
| rs3740999  rs11038294  rs12272467 | *TRIM6* | x | AF-Q9C030-F1  (0.8) | Kidney |
| rs67573252 | *TRIM22* | x | AF-Q8IYM9-F1  (0.87) | Kidney and Immune system |
| rs2342380 | *TRIM34* | 2EGP  (0.82) | --- | Whole blood |
| rs2307111 | *POC5* | x | AF-Q8NA72-F1  (0.85) | Testis and whole blood |
| rs450630 | *SCAND3* | x | AF-Q6R2W3-F1  (0.84) | Testis |
| rs10535646 | *SIDT2* | 7Y68  (0.84) | --- | GIT, kidney, and whole blood |
| rs6578521  rs11822851 | *MMP26* | x | AF-Q9NRE1-F1  (0.81) | Endometrium |
| rs2071348 | *HBBP1* | — | — | Whole blood |
| rs2071348 | *HBD* | 1SHR  (0.84) | --- | Whole blood |
| rs2213170  rs7130110  rs2213169 | Near *HBE1* | 1A9W  (0.85) | --- | Whole blood |
| rs2236794 | *HBG2* | 7QU4  (0.88) | --- | Whole blood |
| **SNP (n=21)** | **Genes (n=11), with known**  **drug interaction** |  |  |  |
| rs12075  rs12074934  rs863002 | *ACKR1** | 7P93  (0.82) | --- | Lung |
| rs10838525  rs11038628  rs12786650  rs57956987 | *TRIM5** | 2ECV  (0.78) | --- | Immune system |
| rs55945048 | *RRM1** | 3HNC  (0.77) | --- | Bone marrow and lymphoid tissue |
| rs1800684 | *AGER** | SD7F  (0.79) | --- | Lung, spleen, and endothelial cell |
| rs2494250 | *FCER1A** | 1F2Q  (0.68) | --- | Immune cells |
| rs2524035 | *HLA-G** | 3KYO  (0.84) | --- | Endocrine |
| rs3135006 | *HLA-DQB1** | 1UVQ  (0.80) | --- | Bone marrow and lymphoid tissue |
| rs2395522 | *HLA-DRB1*/*  *HLA-DQB1** | 5NI9  (0.82) | --- | Lymphoid tissue |
| rs2844806 | *HLA-A** | 3MRG  (0.81) | --- | Bone marrow & lymphoid tissue |
| rs3132946  rs3132940  rs3096702  rs9267898 | *NOTCH4** | 7OR3  (0.77) | --- | Whole blood |
| rs10767695  rs7120828  rs4243966 | *STIM1** | 3TEQ  0.81 | --- | Skeletal muscle  and immune cells |
| *: Indicates that the gene product is an established target of existing medications.  *Italic*:  Indicates protein products for which the 3D structures have been determined by experimental methods.  Gene expression data were obtained from the human Protein Atlas, NCBI, or GTEX. | | | | |
